# Cholesterol and glaucoma: a systematic review and meta‐analysis

**DOI:** 10.1111/aos.14769

**Published:** 2021-01-28

**Authors:** Laura Posch‐Pertl, Monja Michelitsch, Gernot Wagner, Brigitte Wildner, Günther Silbernagel, Gudrun Pregartner, Andreas Wedrich

**Affiliations:** ^1^ Department of Ophthalmology Medical University of Graz Graz Austria; ^2^ Department for Evidence‐based Medicine and Evaluation Danube University Krems Krems Austria; ^3^ University Library Medical University of Vienna Vienna Austria; ^4^ Division of Angiology Department of Internal Medicine Medical University of Graz Graz Austria; ^5^ Division of Cardiology Campus Benjamin Franklin Charité Berlin Berlin Germany; ^6^ Institute for Medical Informatics, Statistics and Documentation Medical University of Graz Graz Austria

**Keywords:** cholesterol, glaucoma, high‐density lipoproteins, low‐density lipoproteins, statins

## Abstract

**Purpose:**

Intraocular pressure is the main risk factor for glaucoma; however, additional risk factors may also matter. This systematic review and meta‐analysis were conducted to summarize the evidence regarding the association of cholesterol parameters (total cholesterol, low‐density lipoprotein (LDL) and high‐density lipoprotein (HDL) levels) and glaucoma.

**Methods:**

Four electronic databases were searched for all publications containing ‘glaucoma’ and one of various forms of ‘cholesterol’ or ‘lipoprotein’. Two independent reviewers screened abstracts and potentially full texts of identified articles for eligibility. Risk of bias was assessed with the Newcastle–Ottawa Scale. A random‐effects meta‐analysis was used to investigate the differences in total cholesterol, LDL and HDL levels between patients with and without glaucoma.

**Results:**

Overall, 29 observational studies were included in the systematic review and 26 reported quantitative information to investigate differences in cholesterol parameters between patients with glaucoma (*N* = 7196) and patients without glaucoma (*N* = 350 441). Patients with glaucoma had significantly higher total cholesterol levels than patients without glaucoma (Mean Difference (MD) 7.9 mg/dl, 95% CI 3.3 to 12.5, p = 0.001) and lower mean HDL levels (MD −2.0 mg/dl, 95% CI: −3.1 to −0.9, p = 0.001). Patients with glaucoma had higher mean LDL levels than patients without glaucoma, albeit not statistically significant (MD 6.1 mg/dl, 95% CI: −4.3 to 16.4, p = 0.251).

**Conclusion:**

This systematic review and meta‐analysis of observational studies found an association of glaucoma and high total cholesterol and low HDL levels, respectively. Although this supports the hypothesis that lipid levels pose an additional risk for glaucoma development, heterogeneity was substantial and causality cannot be presumed from identified observational studies.

## Introduction

Glaucoma is the most frequent cause of irreversible blindness worldwide. The main risk factor is intraocular pressure (IOP). Lowering of IOP may preserve the visual field in patients with ´glaucoma and thus remains the most important treatment strategy in glaucoma (Jonas et al. 2017).

However, in some patients IOP lowering does not seem sufficient to stop progression of visual field loss. Therefore, other risk factors are currently investigated (Roddy 2020).

One of these additional risk factors is hypothesized to be lipid levels as polymorphisms in genes encoding proteins important for lipid metabolism such as ABCA1, GAS7 and ATXN2 have been associated with glaucoma (Wiggs & Pasquale 2017). Since a large case‐control study found that long‐term statin use was associated with a reduced risk of glaucoma interest in lipid levels as an additional risk factor has risen (McGwin et al. 2004). However, statins do not only lower lipid levels, but also seem to have an anti‐inflammatory and consequently neuroprotective effect (Xu et al. 2017). This further obscures the relationship between glaucoma, lipid levels and lipid‐lowering drugs.

The available data on the association between cholesterol and glaucoma are conflicting. Some studies found an association between cholesterol and glaucoma (Kim et al. 2014b), while other studies could not confirm this (Modrzejewska et al. 2015). Therefore, we conducted a systematic review and meta‐analysis to summarize the evidence regarding the association of cholesterol parameters (total cholesterol, low‐density lipoprotein and high‐density lipoprotein levels) and glaucoma.

## Methods

### Registration

We registered our systematic review in International prospective register of systematic reviews (PROSPERO registration number CRD42017067748). The reporting in this publication follows the Preferred Reporting Items for Systematic reviews and Meta‐Analyses (PRISMA) statement (see Table S1).

### Literature search

An experienced medical information specialist (BW) systematically searched the electronic databases: MEDLINE, Embase, Cochrane Central Register of Controlled Trials and Science Citation Index Expanded for all publications from database inception until October 2020. Additionally, all bibliographies of identified articles were scanned to identify potentially relevant manuscripts missed by our search in the databases. Using free term and controlled term formulations the following keywords were searched for in the databases: ‘glaucoma’ AND ‘cholesterol’, ‘glaucoma’ AND ‘low‐density lipoprotein’, ‘glaucoma’ AND ‘high‐density lipoprotein’, ‘glaucoma’ AND ‘dyslipidemia’ and ‘glaucoma’ AND ‘lipoprotein’. We limited our search to articles published in English.

### Study eligibility criteria

All observational studies (cross‐sectional, case‐control, cohort, survey and surveillance reports) reporting the association between glaucoma and cholesterol including cross‐sectional, case‐control, cohort, survey and surveillance reports were included. Studies had to report on adult patients (≥18 years) and had to be published in English.

Abstracts and conference proceedings that are not published in peer‐reviewed journals were not included. Furthermore, any publication without original data for the quantitative analysis was excluded for the quantitative meta‐analysis.

Studies had to ascertain diagnosis of glaucoma by any one or more of the following: fundus photo, fundus examination, retinal nerve fibre layer (RNFL) thickness evaluation, visual field defects, medical records, self‐report and/or glaucoma treatment.

### Study selection

Two reviewers (LP, MM) independently screened references for inclusion. After pilot testing, we performed dual abstract screening based on the eligibility criteria. Included references underwent subsequent dual full‐text review to decide on final inclusion or exclusion of the study. Disagreements were resolved by consensus. The online software ‘Covidence’ (Van der Mierden et al. 2019) was used for abstracts and full‐text screening.

### Data extraction

Two investigators (LP, MM) independently extracted the title, name of authors, year of publication, study design, sample size, type of glaucoma, definition of glaucoma and control patients, demographic data and outcome variables (i.e. total cholesterol, LDL, HDL). These data were recorded in a Microsoft Excel (Microsoft Cooperation) spreadsheet.

### Risk of bias assessment

The Newcastle–Ottawa Scale (NOS) was used to assess potential risk of bias in each individual study (Hartling et al. 2013). The NOS was developed to assess the quality of nonrandomized studies. Two reviewers (LP, MM) independently assessed the included studies. Disagreements were solved by consensus.

### Quantitative synthesis

Differences in total cholesterol, LDL and HDL levels between patients with and without glaucoma were analysed using random‐effects meta‐analysis. We extracted means and standard deviations from the studies whenever available. For one study reporting only the range, the standard deviation was estimated as (max−min)/6 due to a sufficiently large sample size (Wan et al. 2014). For studies with more than one glaucoma study arm (e.g. normal‐tension glaucoma (NTG)), we used the weighted mean and pooled standard deviation to combine the arms. For studies with a healthy control group as well as a control group consisting of patients with PEX syndrome without glaucoma, only the healthy controls were considered. Mean differences (MD) between glaucoma and non‐glaucoma patients are displayed in forest plots together with their 95% confidence intervals (CI). Possible publication bias was assessed using Egger’s linear regression test and Begg’s rank correlation test.

Furthermore, we performed leave‐one‐out analyses to assess the influence of each individual study on the overall results. Heterogeneity was assessed by means of the *I* ^2^ value. We tried to explain the heterogeneity through subgroup analyses by grouping studies into whether or not patients using lipid‐lowering drugs or patients with diabetes were excluded. Finally, we performed meta‐regression analyses to account for metric influential parameters; mean triglyceride levels, age, and BMI of patients as well as percentage of female patients were considered. Weighted means between the glaucoma and control groups were used to obtain one value per study.

All statistical analyses were performed using r version 3.5.1 (R Project for Statistical Computing, Vienna, Austria). In particular, the package ‘meta’ was used.

## Results

**Fig. 1:**
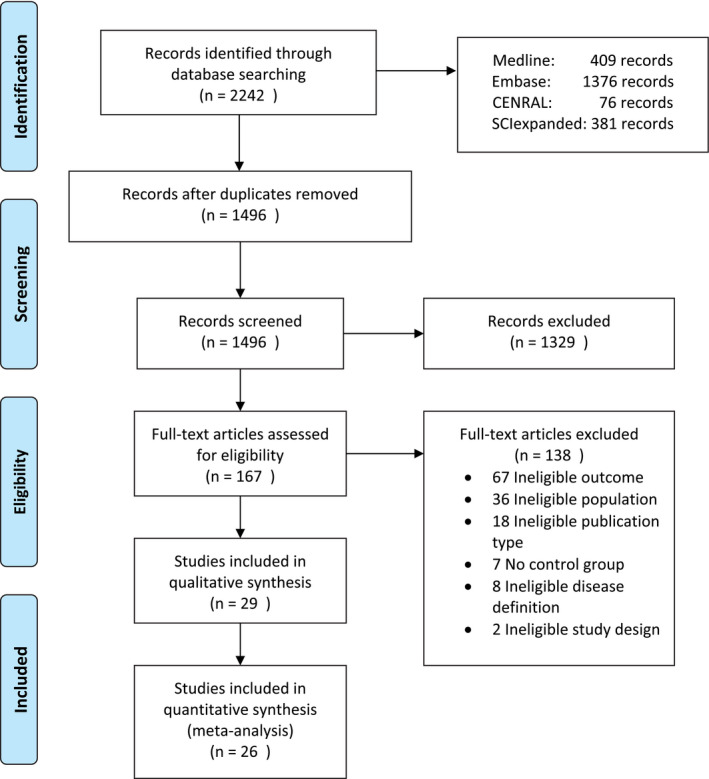
Prisma flow diagram. Adapted from Moher et al. (2009); Liberati et al. (2009). [Colour figure can be viewed at wileyonlinelibrary.com]

Our search yielded 1496 references after removal of duplicates. Figure 1 shows details of the study selection process. After abstract screening, 167 full‐text articles were assessed for eligibility. Most were deemed unfit due to different outcome parameters or study populations as well as ‘ineligible type of publication’. Finally, we included 29 studies in the qualitative synthesis and out of those 26 studies contained relevant information for the quantitative analysis.

### Study characteristics

We included 26 studies investigating the difference in cholesterol levels (i.e. total cholesterol, LDL and/or HDL) between patients with glaucoma (*N* = 7196) and patients without glaucoma (*N* = 350 441). Seven studies included all glaucoma, six specifically NTG, five pseudoexfoliation (PEX) glaucoma, and four studies had two glaucoma arms (e.g. NTG and PEX glaucoma). Controls were recruited at the hospital in four studies, from the community in ten studies, and no recruitment method is given in eight studies (see Table 1 and 2).

**Table 1:** Overview of included studies. Overview of included studies, number of included glaucoma patients and controls.

Study	Number of patients			Target variables		
	Total number	Glaucoma patients	Control patients	Total cholesterol	LDL	HDL
Borger (Borger et al. 2003)	5199	44	5155	1	0	0
Bossuyt (Bossuyt et al. 2015)	63	30	33	1	1	1
Djordjevic‐Jocic (Djordjevic‐Jocic et al. 2014)	291	33	258	1	1	1
Engin (Engin et al. 2010)	191	160	31	1	0	0
Janicijevic (Janicijevic et al. 2017)	80	40	40	1	1	1
Jung, (Jung et al. 2020)	292 523	4970	287 553	1	0	0
Kim (Kim et al. 2016)	4186	124	4062	0	0	1
Kim 126 (Kim et al. 2014a)	18 240	300	17 940	0	0	1
Kim 128 (Kim et al. 2014b)	4095	80	4015	1	0	1
Kurtul (Kurtul et al. 2017)	67	20	47	1	1	1
Lee (Lee et al. 2012)	80	45	35	1	1	1
Mirza (Mirza et al. 2020)	63	21	42	0	0	1
Meier (Meier et al. 2018)	9519	128	9391	1	0	0
Modrzejewska (Modrzejewska et al. 2015)	110	56	54	1	1	1
Ogurel (Ogurel et al. 2016)	54	19	35	1	1	1
Pavljasevic (Pavljasevic & Asceric, 2009)	100	50	50	1	1	1
Rasoulinejad (Rasoulinejad et al. 2015)	200	100	100	1	1	1
Shim (Shim et al. 2015)	167	75	92	1	0	0
Shon (Shon & Sung, 2019)	16 939	561	16 378	1	0	1
Su (Su et al. 2006)	80	40	40	1	1	1
Su (Su et al. 2007)	120	80	40	1	1	1
Türkyilmaz (Turkyilmaz et al. 2014)	50	25	25	1	1	1
Walker (Walker et al. 1976)	4983	63	4920	1	0	0
Yilmaz (Yilmaz et al. 2016)	103	63	40	1	1	1
Yuki (Yuki et al. 2010)	83	43	40	1	0	0
Yüksel (Yuksel et al. 2010)	51	26	25	1	1	1

### Study quality

The NOS score ranged from 1 to 6 showing overall high risk of bias for all 22 studies included in the meta‐analysis.

### Total cholesterol

Twenty‐three studies reported sufficient information to investigate differences in total cholesterol levels in patients with glaucoma (*N* = 6751) and patients without glaucoma (*N* = 328 397). The mean total cholesterol levels strongly varied between studies and ranged from 173.6 mg/dl in the study by Janicijevic et al. (2017) to 254.1 mg/dl in Walker et al (1976).

**Fig. 2:**
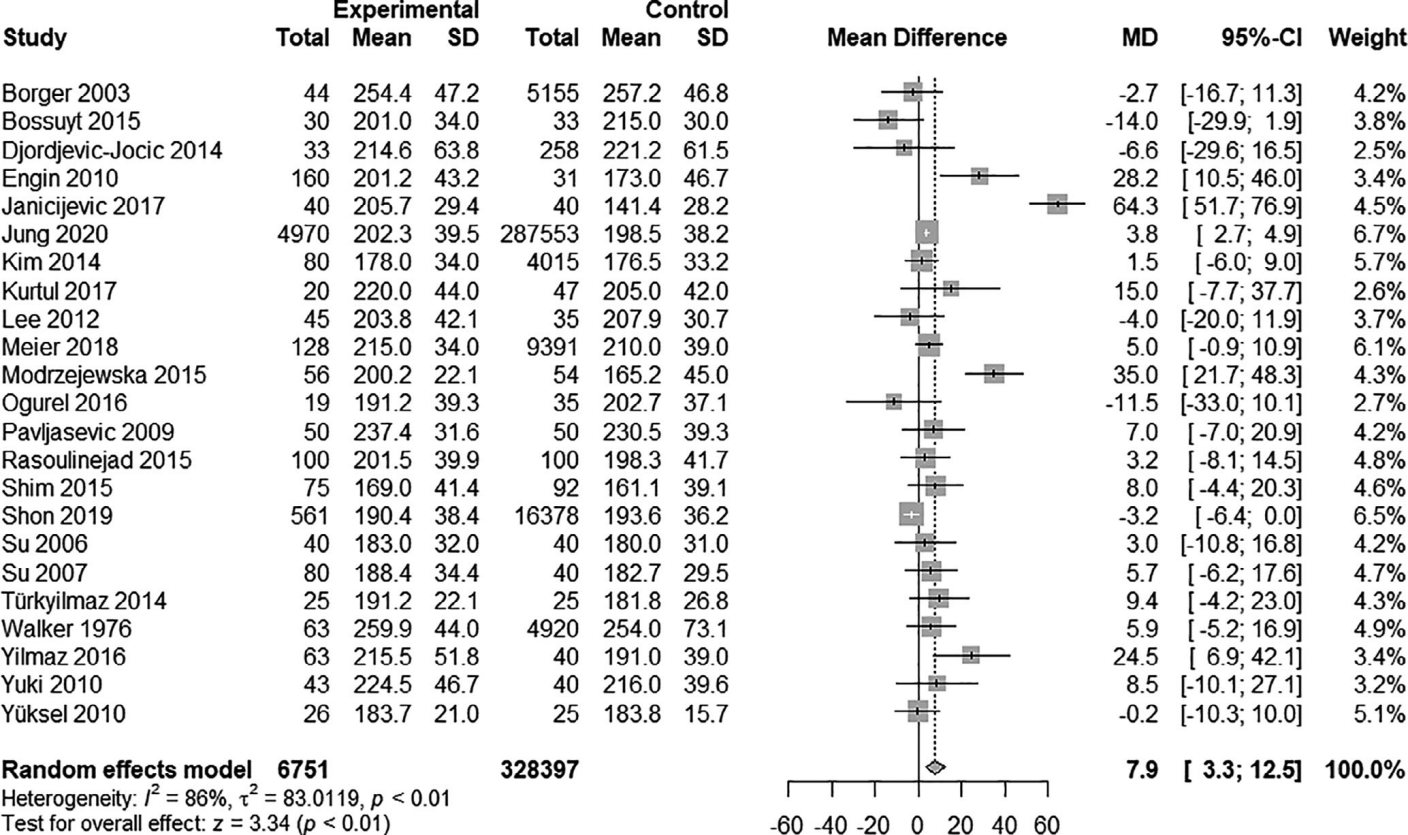
Random‐effects meta‐analysis on total cholesterol level differences between glaucoma patients and controls. The differences are expressed on an absolute scale, that is mg/dl.

Patients with glaucoma had significantly higher total cholesterol levels than patients without glaucoma (MD 7.9 mg/dl, 95% CI 3.3 to 12.5, p = 0.001; Fig. 2). However, there was substantial heterogeneity (*I* ^2^ = 86%, p < 0.01).

In the Baujat plot (see Fig. S1), one study seemed largely to contribute to overall heterogeneity (Janicijevic et al. 2017). When omitting this study, results remained significant (95% CI 1.4 to 8.2, p = 0.006). The study by Shon & Sung (2019) seemed to influence overall results greatly. Again, results remained significant (95% CI 3.4 to 14.0, p = 0.001) after omitting this study (Table 2).

**Table 2:** Overview of inclusion and exclusion criteria.

Study	Glaucoma type	Glaucoma definition	Control type	Origin of study	Age (Mean)		Female (%)		BMI* (Mean)		Lipid‐lowering medication (%)		Diabetes (%)		CVD^†^ (%)		Smokers (ever) (%)	
					G^‡^	C	G	C	G	C	G	C	G	C	G	C	G	C
Borger (Borger et al. 2003)	OAG	Glaucomatous optic neuropathy and glaucomatous visual field defect	Population within Rotterdam Study	Netherlands	72	69	48	60	26	26	n/d	n/d	10.1	14	3.2	2.9	59.6	47.7
Bossuyt (Bossuyt et al. 2015)	NTG	Neuroretinal rim loss and typical visual field defect and normal IOP < 21mmHg	Local community	Netherlands	65	67	77	76	26	26	27	21	Excl^§^	excl	excl	excl	0^¶^	3^¶^
Djordjevic‐Jocic (Djordjevic‐Jocic et al. 2014)	Glaucoma	Definition by Foster (Foster et al. 2002)	Outpatient Department (Nephrology and Haemodialysis)	Serbia	66	64	61	45	28	27	n/d	n/d	46	32	n/d	n/d	21	34
Engin (Engin et al. 2010)	Glaucoma	No clear definition given	n/d	Turkey	51	45	66	48	n/d	n/d	n/d	n/d	excl	excl	excl	excl	n/d	n/d
Janicijevic (Janicijevic et al. 2017)	PEX glaucoma	Elevated IOP and optic disc glaucomatous changes and functional failure of the visual field	n/d	Serbia	n/d	n/d	n/d	n/d	n/d	n/d	n/d	n/d	n/d	n/d	n/d	n/d	n/d	n/d
Jung, (Jung et al. 2020)	Incident Glaucoma	No clear definition given	Health Insurance (KNHIS**)	South Korea	n/d	n/d	48	51	24	24	n/d	n/d	21	10	n/d	n/d	25	28
Kim (Kim et al. 2016)	Glaucoma	Definition by Foster (Foster et al. 2002)	Survey (KNHANES^††^)	South Korea	50	40	35	51	n/d	n/d	n/d	n/d	excl	excl	n/d	n/d	67	45
Kim 126 (Kim et al. 2014a)	NTG	IOP level below 22 mmHg and normal open anterior chamber angle and the presence of glaucomatous optic nerve head change and corresponding visual field change	Glaucoma Screening Programme (Seoul National University Hospital)	South Korea	54	53	32	43	24	24	n/d	n/d	n/d	n/d	n/d	n/d	n/d	n/d
Kim 128 (Kim et al. 2014b)	NTG	Definition by Foster (Foster et al. 2002)	Survey (KNHANES^††^)	South Korea	32	31	50	58	23	23	n/d	n/d	4	1	n/d	n/d	n/d	n/d
Kurtul (Kurtul et al. 2017)	PEX glaucoma	Presence of exfoliation material in the anterior chamber and IOP over 21 mmHg and open anterior chamber angle and visual field changes and optic nerve changes	Outpatient Department	Turkey	71	68	35	57	n/d	n/d	excl	excl	25	4	15	4	n/d	n/d
Lee (Lee et al. 2012)	NTG	Open anterior chamber angles and IOP < 22 mmHg and glaucomatous optic disc cupping and visual field defects	Glaucoma clinic	South Korea	54	52	51	49	n/d	n/d	excl	excl	excl	excl	excl	excl	n/d	n/d
Mirza (Mirza et al. 2020)	PEX glaucoma	Presence of PEX material and IOP ≥ 21 mmHg and open anterior chamber angle and presence of cup to disc ratio > 0.5 and glaucomatous changes in the optic disc and in the visual field	Ophtalmology Department	Turkey	68	67	48	43	n/d	n/d	excl	excl	excl	excl	excl	excl	excl	excl
Meier (Meier et al. 2018)	Incident Glaucoma	No clear definition given	Preventive medical examination (Aerobics Center Longitudinal Study)	Unites States of America	54	50	19	19	26	26	n/d	n/d	3	4	2	1	52	40
Modrzejewska (Modrzejewska et al. 2015)	Glaucoma patients	Treated with antiglaucomatous beta‐blocker topical drops and typical glaucomatous optic neuropathy changes and visual field lesions and intraocular pressure elevation	Healthy volunteers	Poland	68	68	n/d	n/d	n/d	n/d	excl	excl	excl	excl	excl	excl	excl	excl
Ogurel (Ogurel et al. 2016)	PEX glaucoma	Presence of exfoliation materials and raised IOP	Outpatient Department	Turkey	65	65	47	51	31	33	n/d	n/d	excl	excl	excl	excl	excl	excl
Pavljasevic (Pavljasevic & Asceric, 2009)	POAG	IOP eye > 20mmHg and visual field changes and open angle	Ophthalmology Department	Bosnia and Herzegovina	59	59	66	58	n/d	n/d	n/d	n/d	n/d	n/d	n/d	n/d	n/d	n/d
Rasoulinejad (Rasoulinejad et al. 2015)	POAG	Definition by Foster (Foster et al. 2002)	n/d	Iran	62	60	66	58	24	25	n/d	n/d	n/d	n/d	n/d	n/d	n/d	n/d
Shim (Shim et al. 2015)	NTG	Disc haemorrhage and open angle and glaucomatous optic disc abnormality and glaucomatous visual field loss and IOP < 21mm Hg	Outpatient Department	South Korea	56	59	40	34	25	25	n/d	n/d	n/d	n/d	n/d	n/d	n/d	n/d
Shon (Shon & Sung, 2019)	Glaucoma	Definition by Foster (Foster et al. 2002)	Survey (KNHANES^††^)	South Korea	63	58	51	57	24	24	11	8	18	11	n/d	n/d	44	41
Su (Su et al. 2006)	NTG	Untreated IOP < 22 mm Hg and open anterior chamber angles and glaucomatous optic disc cupping and characteristic optic nerve‐related visual field loss	Routine physical check‐up	Taiwan	50	49	38	50	24	25	n/d	n/d	excl	excl	excl	excl	n/d	n/d
Su (Su et al. 2007)	NTG and POAG	Untreated IOP < 22 mm Hg, respectively IOP > 21mmHg and open anterior chamber angles and glaucomatous optic disc cupping and characteristic optic nerve‐related visual field loss	Routine physical check‐up	Taiwan	51	49	40	48	24	25	excl	excl	excl	excl	excl	excl	n/d	n/d
Türkyilmaz (Turkyilmaz et al. 2014)	PEX glaucoma	No clear definition given	n/d	Japan	65	64	68	56	25	25	excl	excl	excl	excl	excl	excl	excl^¶^	excl^¶^
Walker (Walker et al. 1976)	NTG and POAG	No clear definition given	n/d	United Kingdom	n/d	n/d	30	43	n/d	n/d	n/d	n/d	excl	excl	9	18	53^¶^	32^¶^
Yilmaz (Yilmaz et al. 2016)	NTG and PEX glaucoma	IOP ≤ 21 mmHg, respectively exfoliation material nad IOP > 21 mmHg and glaucomatous changes in the optic disc and in the visual field	n/d	Turkey	60	55	69	55	27	27	n/d	n/d	21	5	n/d	n/d	excl^¶^	excl^¶^
Yuki (Yuki et al. 2010)	Incident NTG	Open anterior chamber angles and glaucomatous optic disc cupping and the presence of a nerve fibre layer defect and visual field defect	Refractive Check‐Up	Japan	59	62	63	65	22	23	n/d	n/d	excl	excl	excl	excl	excl	excl
Yüksel (Yuksel et al. 2010)	PEX glaucoma	Glaucomatous optic neuropathy and visual field damage and PEX material and an intraocular pressure of > 22 mmHg without treatment	Ophthalmology Department	Turkey	66	65	31	48	27	28	excl	excl	excl	excl	excl	excl	excl	excl

* Body Mass Index.

^†^ Cardiovascular disease.

^‡^ Glaucoma patients (G) and control patients (C).

^§^ Excluded in resepective study population.

^¶^ Numbers of active smokers.

** Korean Health Insurance System.

^††^ Population‐based cross‐sectional survey performed by the Korea Centers for Disease Control and Prevention.

To detect publication bias, Eggers test and Beggs test were performed. Both were not significant (p = 0.271, respectively 0.653) suggesting unbiased results.

Subgroup analysis revealed that studies excluding patients taking lipid‐lowering drugs had a smaller mean absolute difference of total cholesterol levels between glaucoma patients and controls. In detail, studies excluding patients on lipid‐lowering drugs showed an MD of 3.7 mg/dl (95% CI −2.3 to 9.6) between glaucoma and control patients, and studies including patients on lipid‐lowering drugs an MD of 8.8 mg/dl (95% CI 3.4 to 14.3).

Another subgroup analysis showed that studies excluding diabetics also had a smaller mean absolute difference. In detail, studies excluding diabetics had a MD of 4.6 mg/dl (95% CI −0.5 to 9.7) between glaucoma patients and controls, studies with diabetics a MD of 5.6mg/dl (95% CI −2.1 to 13.4) and studies with no information of diabetics 13.9 mg/dl (95% CI −5.6 to 33.3).

In meta‐regression analysis, triglycerides (p = 0.277), age (p = 0.854), BMI (p = 0.518) and gender (p = 0.745) did not appear to modify the association between glaucoma and total cholesterol, but were also not able to explain the large heterogeneity.

### Low‐density lipoproteins

Fourteen studies provided information to investigate the difference in LDL levels in patients with glaucoma (*N* = 627) and patients without glaucoma (*N* = 822). The mean LDL levels strongly varied between studies and ranged from 104.1 mg/dl in the study by Rasoulinejad et al. (2015) to 155.5 mg/dl in Pavljasevic & Asceric (2009).

**Fig. 3:**
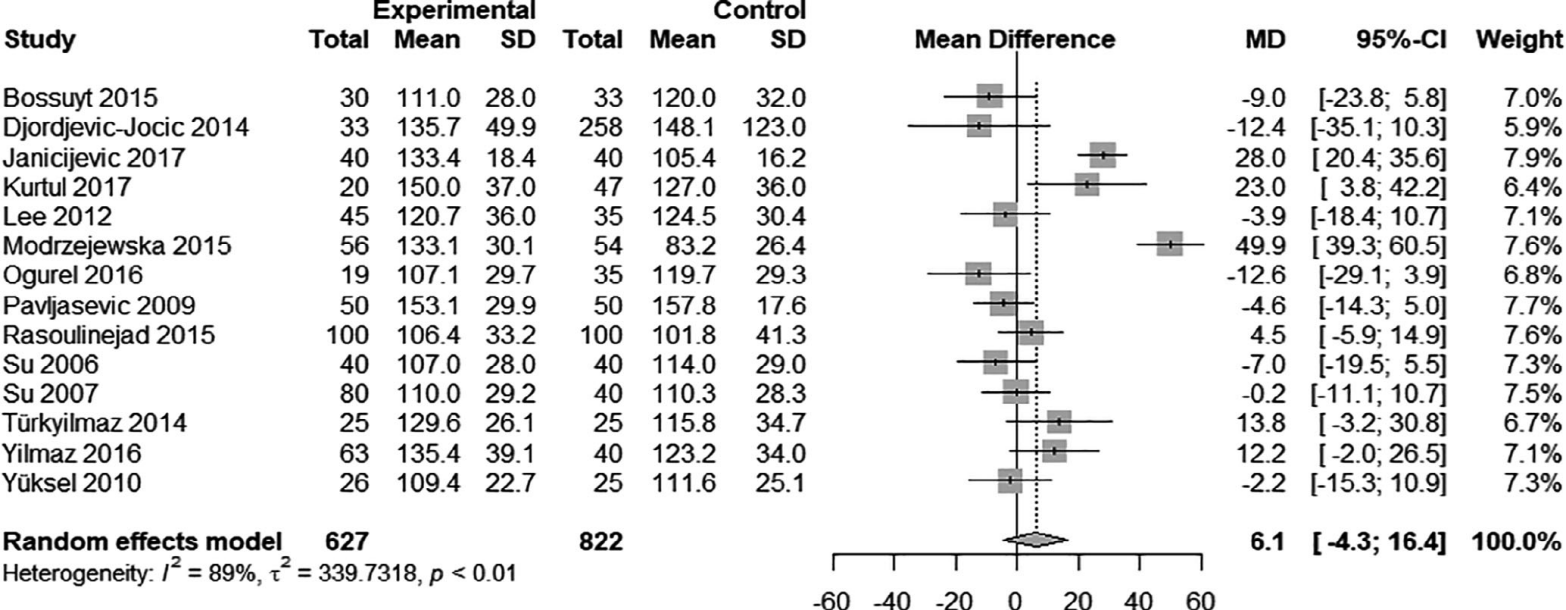
Random‐effects meta‐analysis on LDL level differences between glaucoma patients and controls. The differences are expressed on an absolute scale, that is mg/dl.

Patients with glaucoma had higher mean LDL levels than patients without glaucoma, albeit not statistically significant (MD 6.1 mg/dl, 95% CI: −4.3 to 16.4, p = 0.251; Fig. 3). Again, there was substantial heterogeneity (*I* ^2^ = 89%, p < 0.01).

In the Baujat plot (see Fig. S2) two studies, two studies seemed to largely contribute to overall heterogeneity and influence overall results (Modrzejewska et al. 2015; Janicijevic et al. 2017), when omitting these results were still not significant (95% CI −6.3 to 14.7, p = 0.431, respectively 95% CI −5.5 to 10.7, p = 0.528). Regarding publication bias, Eggers test and Beggs test were not significant (p = 0.153, respectively p = 0.87).

Subgroup analysis showed that studies excluding patients on lipid‐lowering drugs had a smaller mean absolute difference (MD 4.3, 95% CI: −4.6 to 13.2) than studies including patients on lipid‐lowering drugs (MD 6.1, 95% CI: −8.6 to 20.8).

### High‐density lipoproteins

Nineteen studies contained information to investigate the difference in HDL levels in patients with glaucoma (*N* = 1713) and patients without glaucoma (*N* = 43 259). The mean HDL levels strongly varied between studies and ranged from 41.1 mg/dl in the study by Modrzejewska et al. (2015) to 72.1 mg/dl in Bossuyt et al. (2015).

**Fig. 4:**
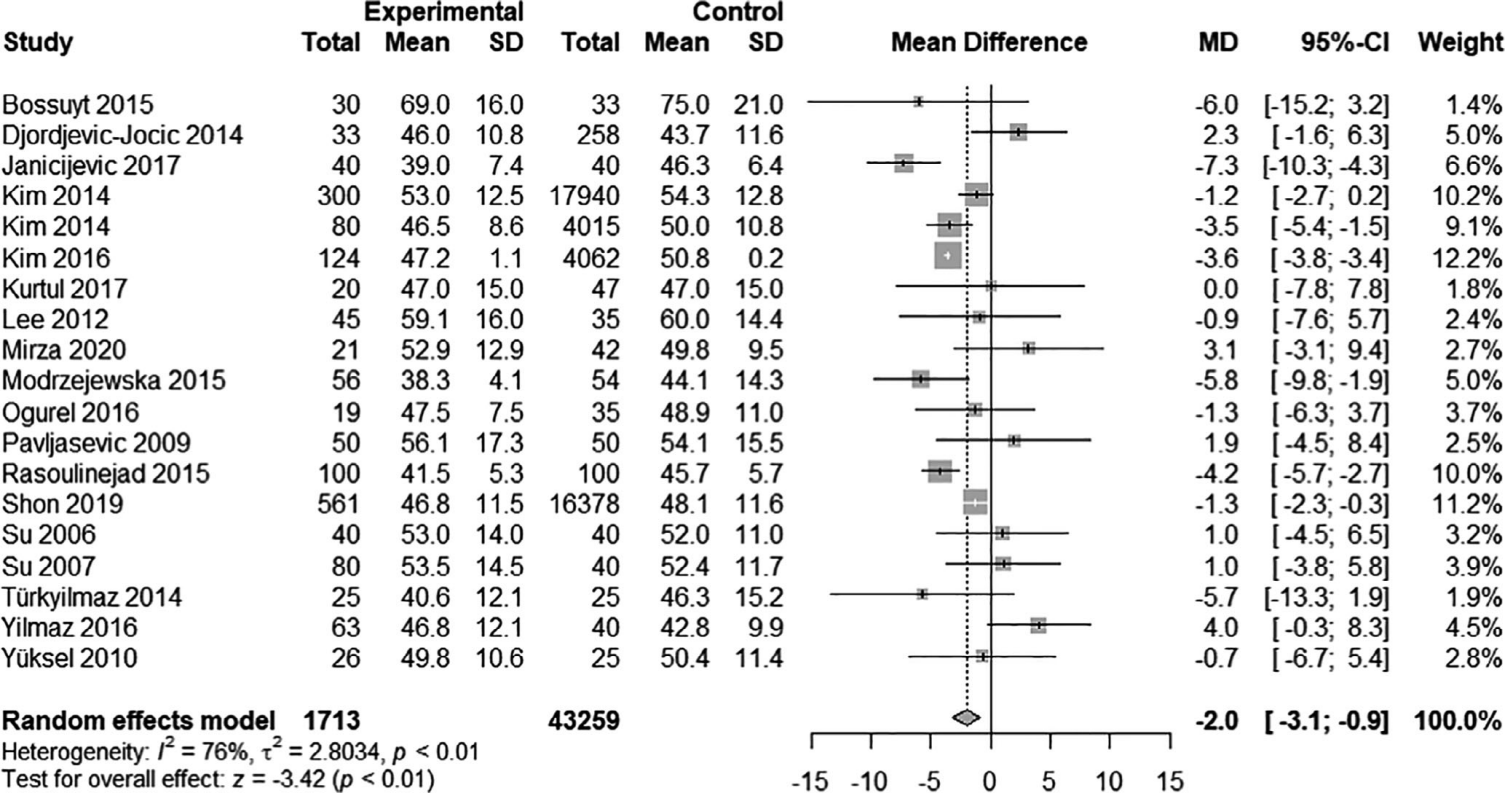
Random‐effects meta‐analysis on HDL level differences between glaucoma patients and controls. The differences are expressed on an absolute scale, that is mg/dl.

Patients with glaucoma had lower mean HDL levels (MD −2.0 mg/dl, 95% CI: −3.1 to −0.9, p = 0.001; Fig. 4) than patients without glaucoma and the heterogeneity was substantial (*I* ^2^ = 69%, p < 0.01).

One study in particular (Kim et al. 2016) had an influence on overall results (see Fig. S3). Results remained significant after omission of this study (95% CI −3.0 to −0.4, p = 0.013). Three studies contributed largely to overall heterogeneity (Kim et al. 2014b; Yilmaz et al. 2016; Shon & Sung, 2019). Again, after omitting this study, results remained significant (95% CI −3.0 to −0.4, p = 0.004; 95% CI −3.3 to −0.8, p = 0.001, respectively 95% CI −3.4 to −1.2, p < 0.001). For HDL results Eggers test was significant (p = 0.003), while Beggs test was not (p = 0.753).

Subgroup analysis showed that studies excluding patients on lipid‐lowering drugs had a smaller mean absolute difference (MD −0.7, 95% CI: −3.5 to 2.1) than studies including patients on lipid‐lowering drugs (MD −2.5, 95% CI: −3.8 to −1.1).

In meta‐regression, triglycerides modified the association between HDL and glaucoma (p = 0.007). Age (p = 0.291), BMI (p = 0.235) and gender (p = 0.238) did not modify the association between HDL and glaucoma.

## Discussion

This systematic review and meta‐analysis of observational studies found that patients with glaucoma had higher mean total cholesterol levels and lower HDL levels than patients without glaucoma, whereas there was no significant difference regarding LDL. This may support the conjecture that total cholesterol levels and HDL levels pose an additional risk factor for glaucoma. However, no causality can be drawn from observational studies.

The studies on the association between glaucoma and lipid levels included in our systematic review found different results, which resulted in high heterogeneity across studies in the meta‐analyses. There are several possible reasons for this. First of all, different types of glaucoma were included. Secondly, there were differences in inclusion criteria among the individual studies. Furthermore, the exclusion criteria of individual studies differed greatly. For example, some studies excluded patients with lipid‐lowering drugs, while others included them or did not report on the use of lipid‐lowering medications (see Table 1).

Subgroup analysis showed that studies excluding patients on lipid‐lowering drugs showed smaller mean differences in total cholesterol levels and HDL levels between glaucoma and control patients. It has been suggested that statin use reduces the incidence of glaucoma (McCann et al. 2016). Therefore, confounding by indication is a possibility in this meta‐analysis and our findings may be exaggerated. Otherwise it could be that patients taking lipid‐lowering drugs have high cholesterol and thus studies excluding these patients had smaller mean differences. This would support our notion that cholesterol plays a role in the development of glaucoma.

Statin use has been shown to protect against glaucoma development and progression. It seems that the beneficial effect of statins is associated less with lowering lipid levels and more with other properties of statins such as possible anti‐inflammatory properties (Talwar et al. 2017). In this meta‐analysis, LDL levels were not associated with glaucoma, which might support the concept that statin lowers the glaucoma risk via other mechanism than lowering lipid levels. As our study found that HDL and total cholesterol levels were associated with glaucoma, it might be interesting to see whether statins, which additionally increase HDL levels such as pitavastatin (Pirillo & Catapano, 2017), may be even better in reducing glaucoma development than statins pre‐eminently reducing LDL.

Topical beta‐blockers seem to lower HDL (Stewart et al. 1999) (Yamamoto et al. 1996). This was confirmed in the Blue Mountains Eye study, although the adverse effect on HDL was seen exclusively in men (Mitchell et al. 2000). Most of the included studies did not report on topical treatment used and thus no meta‐regression could be performed. It may be assumed that a large number of patients use topical beta‐blockers and the effect seen in this meta‐analysis is due to this side effect.

We want to mention several limitations of this meta‐analysis. Firstly, we only included observational studies. Observational data cannot prove causality. Further, there is some evidence that cholesterol influences IOP (Wang et al. 2019). Therefore, this study cannot answer whether cholesterol leads to glaucoma via IOP or whether this association is independent of IOP. Secondly, there was a large heterogeneity between studies concerning types of studies, inclusion and exclusion criteria and the selection of the control group. Additionally, Eggers test was significant in our meta‐analysis on HDL and glaucoma suggesting publication bias. Thirdly, statin use is a potential confounder in this study, which could not be fully accounted for. No meta‐regression could be performed due to the small number (*n* = 2) of studies reporting the percentage of glaucoma and control patients taking lipid‐lowering drugs. Another potential bias are errors in the diagnosis of glaucoma, that is glaucoma cases were misidentified as normal and vice‐versa. Finally, our results were statistically significant. However, mean absolute differences in total cholesterol and HDL were still small (MD 9.2 mg/dl, respectively −2.3 mg/dl) and thus, of unclear clinical relevance.

In conclusion, this meta‐analysis of observational studies found an association of glaucoma and high total cholesterol and low HDL levels, respectively. Although this supports the hypothesis that lipid levels pose an additional risk for glaucoma development, we were unable to explain the large heterogeneity and causality can generally not be presumed from identified observational studies.

## Supporting information


**Figure S1.** Baujat‐ and Funnel‐Plot for meta‐analysis on total cholesterol and glaucoma.

**Figure S2.** Baujat‐ and Funnel‐Plot for meta‐analysis on LDL and glaucoma.

**Figure S3.** Baujat‐ and Funnel‐Plot for meta‐analysis on HDL and glaucoma.

**Table S1.** PRISMA Checklist. Adapted from Moher et al. (2009), Liberati et al. (2009).
