# Supplementary figures and images for: Cholesterol and glaucoma: a systematic review and meta‐analysis
Source: Acta Ophthalmol. 2021 Jan 28;100(2):148–58. doi: 10.1111/aos.14769 (PMC9292534; doi:10.1111/aos.14769)

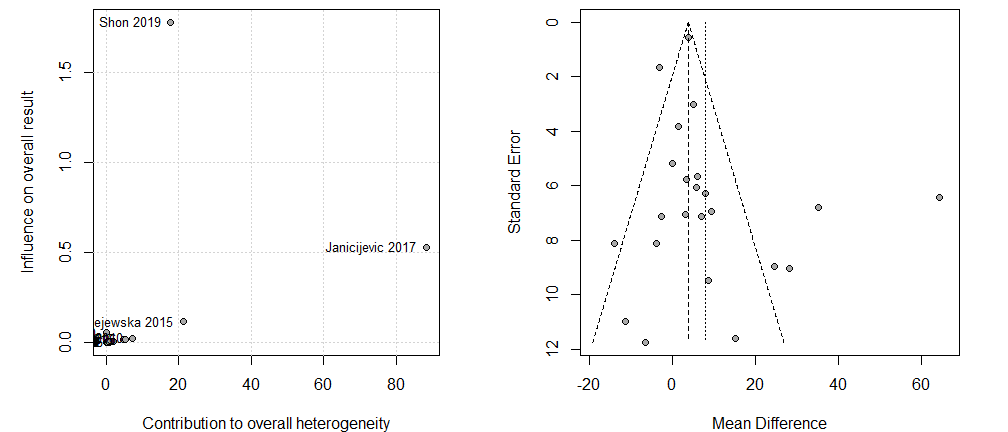
**Figure S1. Baujat- and Funnel-Plot for meta-analysis on total cholesterol and glaucoma.**

Supplement: Supplementary file 1 — Figure S1. Baujat‐ and Funnel‐Plot for meta‐analysis on total cholesterol and glaucoma. [file AOS-100-148-s002.docx]

**Figure S2. Baujat- and Funnel-Plot for meta-analysis on LDL and glaucoma.**


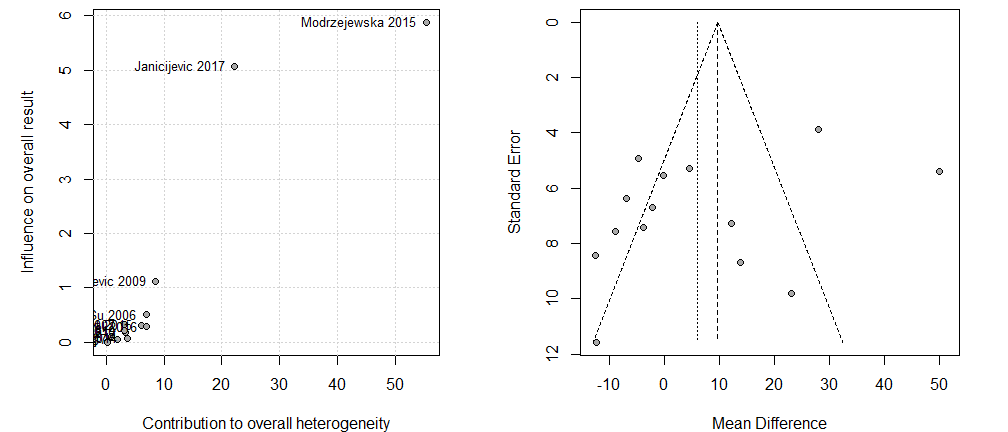

Supplement: Supplementary file 2 — Figure S2. Baujat‐ and Funnel‐Plot for meta‐analysis on LDL and glaucoma. [file AOS-100-148-s003.docx]

**Figure S3. Baujat- and Funnel-Plot for meta-analysis on HDL and glaucoma.**


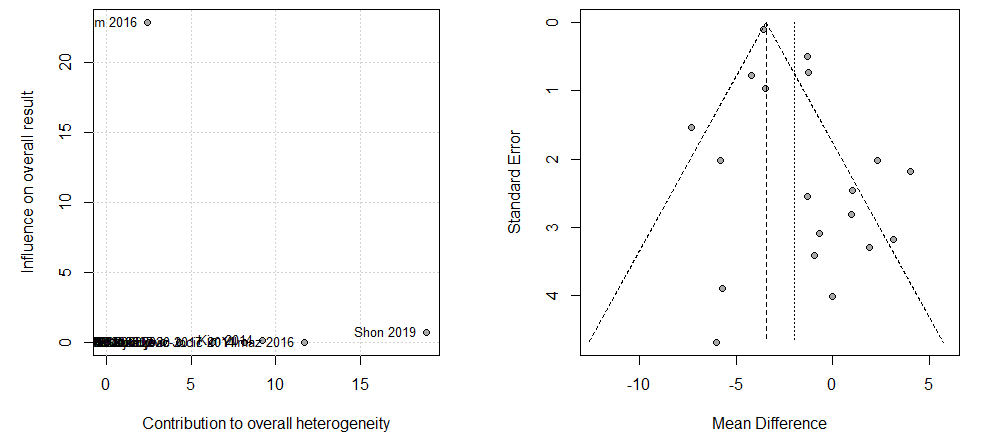

Supplement: Supplementary file 3 — Figure S3. Baujat‐ and Funnel‐Plot for meta‐analysis on HDL and glaucoma. [file AOS-100-148-s001.docx]
